# Early Pregnancy-Associated Plasma Protein A Concentrations Are Associated With Third Trimester Insulin Sensitivity

**DOI:** 10.1210/jc.2017-00272

**Published:** 2017-03-13

**Authors:** Clive J. Petry, Ken K. Ong, Ieuan A. Hughes, Carlo L. Acerini, Jan Frystyk, David B. Dunger

**Affiliations:** 1 Department of Paediatrics, University of Cambridge, Cambridge CB2 0QQ, United Kingdom; 2 Medical Research Council Epidemiology Unit, University of Cambridge, Cambridge CB2 0QQ, United Kingdom; 3 Medical Research Laboratory, Department of Clinical Medicine, Aarhus University, 8000 Aarhus, Denmark; 4 Institute of Metabolic Science, University of Cambridge, Cambridge CB2 0QQ, United Kingdom

## Abstract

**Context:**

First or early second trimester pregnancy-associated plasma protein A (PAPP-A) concentrations have previously been shown to be lower in women who subsequently develop gestational diabetes mellitus (GDM) and gestational hypertension.

**Objective:**

We therefore sought to investigate why circulating PAPP-A concentrations are related to the subsequent risk of GDM and gestational hypertension.

**Patients, Design, and Setting:**

We measured serum PAPP-A concentrations around week 15 of pregnancy and related these to indices derived from week 28 oral glucose tolerance tests and blood pressures across pregnancy in the Cambridge Baby Growth Study cohort.

**Results:**

Increased PAPP-A concentrations were associated with reduced GDM risk [odds ratio 0.623 (0.453, 0.856), *P* = 3.5 × 10^−3^, n = 777] and reduced mean arterial blood pressures (*β* = −0.202 to −0.177, *P* = 1.7 to 6.9 × 10^−3^, n = 347 to 355). They were also negatively associated with week 28 fasting (*β* = −0.149, *P* = 6.6 × 10^−4^, n = 777) and 60-minute (*β* = −0.188, *P* = 1.5 × 10^−5^, n = 777) oral glucose tolerance test glucose concentrations. These associations were underpinned by the strong associations between increased week 15 PAPP-A concentrations and decreased week 28 insulin resistance (homeostasis model assessment of insulin resistance: *β* = −0.319, *P* = 1.7 × 10^−13^, n = 768), as well as increased insulin secretion relative to insulin sensitivity (insulin disposition index: *β* = 0.202, *P* = 6.5 × 10^−6^, n = 731).

**Conclusions:**

These results suggest that links between PAPP-A concentrations in early pregnancy and subsequent glucose concentrations and blood pressures may be mediated by changes in insulin sensitivity (and secretion).

## Teaser

Pregnancy-associated plasma protein A concentrations, measured at the start of the second trimester, were associated with subsequent insulin sensitivity, blood pressure, and gestational diabetes.

Pregnancy associated plasma protein A (PAPP-A) is produced by the placenta in pregnancy and is one of only four placentally derived proteins found in the maternal circulation in high concentrations (1). It largely circulates as a heterotetramer consisting of two PAPP-A subunits covalently bound to two subunits of the preform of eosinophil major basic protein (2). The principal function of PAPP-A as a metalloproteinase of the metzincin superfamily appears to be the cleavage of circulating insulin-like growth factor (IGF) binding protein (IGFBP)-4, although there are suggestions that PAPP-A may also be involved in the cleavage of IGFBP-2 (3) and IGFBP-5 (4). It is only the uncomplexed, dimeric form of PAPP-A, the proportion of which varies throughout pregnancy but is ∼1% of circulating concentrations, that displays this proteolytic activity (5). Whichever binding protein uncomplexed PAPP-A cleaves it would appear to have a role in regulating IGF bioavailability in pregnancy (6). This is important, as the IGF axis plays a critical role in fetal growth and placental growth and function during pregnancy (7).

Measurement of circulating PAPP-A concentrations is used, along with nuchal translucency scanning and free *β*-human chorionic gonadotropin concentration measurement, in first trimester screening for fetal chromosomal abnormalities, including Down syndrome, Patau syndrome, and Edward syndrome (trisomies 21, 13, and 18, respectively). In pregnancy, circulating PAPP-A concentrations are also influenced by such factors as gestational age (increasing in a curvilinear fashion with gestational age), maternal weight (decreasing with increasing weight) and height (increased with increasing height), ethnicity (higher in women of Afro-Caribbean, East Asian, and South Asian racial origin), method of conception (decreased in first trimester and raised in the third trimester with *in vitro* fertilization), parity (decreased in parous women), and smoking status (decreased in smokers) (8). Additionally, they appear to be decreased by pre-existing type 1 and type 2 (8) diabetes. A number of studies have found associations between reduced circulating PAPP-A concentrations early in pregnancy and the subsequent development of gestational diabetes mellitus (GDM) (9–16), although these are not uniform occurrences (17, 18). Where such associations have been found, studies have tended not to investigate mechanisms that may be underpinning these associations. Other studies have found associations between reduced circulating PAPP-A concentrations early in pregnancy and the development of gestational hypertension (13, 19) or pre-eclampsia later in pregnancy (20, 21), conditions that are commonly linked with GDM. As both GDM and gestational hypertension can affect the baby’s birth weight, early pregnancy circulating PAPP-A concentrations have been related to birth weight in several studies (16, 18, 22–29). In this study, we attempted to extend the relationships of early pregnancy PAPP-A concentrations with the adverse conditions of pregnancy to other markers of impaired glucose tolerance derived from the oral glucose tolerance test (OGTT) and high blood pressures. We hypothesized that the relationships were mediated by changes in IGF bioactivity.

## Research Design and Methods

### Cambridge Baby Growth Study

The Cambridge Baby Growth Study (prospective and longitudinal) recruited mothers attending ultrasound clinics during early pregnancy at the Rosie Maternity Hospital (Cambridge, UK) between 2001 and the present. Blood samples were drawn at recruitment (and centrifuged, with the serum separated and stored at −80°C until analysis). There were two main phases of recruitment for the Cambridge Baby Growth Study. Phase I ran from 2001 until 2009, from which the samples used in this study were obtained. Blood samples were collected on average at week 15 of pregnancy at the booking clinic (n = 821). Table 1 shows the clinical characteristics of the study participants who had blood samples taken. At 28 weeks of gestation, 1074 of the mothers underwent a 75-g OGTT after an overnight fast. Venous blood was collected after fasting and at 60 minutes after the glucose load for the measurement of plasma glucose concentrations (using a routine glucose oxidase–based method). Capillary whole-blood glucose concentrations were measured at 0, 30, 60, 90, and 120 minutes using an Abbott Freestyle mini kit (Abbott Diagnostics, Maidenhead, UK). Routine blood pressure measurements during three time points in pregnancy were documented from hospital notes (30). The baby’s birth weights were recorded by hospital midwives and retrieved from the hospital notes. In this cohort, 96.9% of the offspring were white, 0.8% were of mixed race, 0.6% were black (African or Caribbean), 0.8% were Oriental, and 0.9% were Indo-Asian.

**Table 1.** **Clinical Characteristics of the Cambridge Baby Growth Study Participants Who Had Week 15 Serum PAPP-A Concentrations Measured**

Demographic	
N	821
Maternal age at birth of baby, y	33.2 (32.9, 33.5)
	(n = 689)
Parity	1.7 (1.6, 1.8)
	(n = 779)
Prepregnancy BMI, kg/m^2^	24.1 (23.9, 24.5)
	(n = 643)
Unadjusted birth weight of baby, kg	3.498 (3.462, 3.535)
	(n = 779)
Percentage giving birth to males, %	52.6
Gestational age of offspring at delivery, decimal wk	39.9 (39.8, 40.0)
	(n = 782)
Percentage that smoked at any point during pregnancy	4.1
Percentage that developed GDM	8.9
Percentage that developed gestational hypertension	6.0

Data are mean (95% confidence intervals) or percentages.

Abbreviation: BMI, body mass index.

### Disease diagnostic thresholds

The Cambridge Baby Growth Study participants were divided into cases and controls for GDM according to the International Association of Diabetes in Pregnancy Study Group criteria (31) using the OGTT fasting and 60-minute plasma glucose concentrations. Its prevalence in the Cambridge Baby Growth Study was 10.2%. Evidence of gestational hypertension was sought from the hospital notes (defined using the inclusion of a diagnosis of pre-eclampsia, gestational hypertension, or pregnancy-induced hypertension). Alternatively, the National Institute for Health and Care Excellence criteria for defining gestational hypertension (blood pressure measurements in the second half of pregnancy ≥ 140 mm Hg systolic or 90 mm Hg diastolic blood pressure in women without chronic hypertension) (32) were used, with the exception that for our study evidence of gestational hypertension was accepted when the blood pressure cut-offs were exceeded at one reading rather than at least two. Its prevalence in the Cambridge Baby Growth Study was 6.1% (30).

### Cohort subgroups

The 821 serum samples collected early in the second trimester of pregnancy were ordered according to their PAPP-A concentrations. The 48 samples with the lowest PAPP-A concentrations formed the “low PAPP-A” group, and the 48 samples with the highest PAPP-A concentrations formed the “high PAPP-A” group. Bioactive IGF was estimated in these subgroups.

### Ethical approval

The Cambridge Baby Growth Study was approved by the local ethics committee (Addenbrooke’s Hospital, Cambridge, UK). Written informed consent was obtained from all of the mothers who took part in this study.

### Circulating hormone concentration measurements

PAPP-A was measured by time-resolved fluoroimmunoassay (AutoDELFIA; Perkin Elmer, Seer Green, UK). The minimum detection limit of this assay was 22.5 mg/L (5 mU/L). The intra-assay coefficient of variation (CV) was <8% and the interassay CV was <10% throughout. Insulin was measured by enzyme-linked immunosorbent assay using a commercial kit (DSL, London, UK). Sensitivity was 0.26 mU/L (1.6 pmol/L). Intra-assay CVs were 4.4% and 5.1% at 10.4 mU/L (62 pmol/L) and 35.9 mU/L (215 pmol/L), and equivalent interassay CVs were 8.7% and 2.9%; this assay has no cross-reactivity with proinsulin at levels up to 9.1 µg/L (1000 pmol/L). Bioactive IGF is a cell-based measurement, which assesses the ability of serum IGF-I and IGF-II to phosphorylate the IGF-I receptor (IGF1R) *in vitro*, using human embryonic cells transfected with cDNA of the human *IGF1R* gene (33). The serum signal is compared with a serial IGF-I dilution and expressed in micrograms per liter. The detection of phosphorylated IGF1R in crude cell lysates was performed using a commercial kit from R&D Systems (Abingdon, UK; catalog no. DYC 1770E). Sensitivity was <0.08 µg/L. The intra-assay CV averages 6% for the signals and 12% for the corresponding concentrations; the long-term interassay CV is 20%.

### Calculations

Mean arterial blood pressure was calculated as the sum of the systolic and twice the diastolic blood pressures, all divided by three. Insulin sensitivity was estimated using the homeostasis model assessment (HOMA), calculated using the week 28 circulating glucose and insulin concentrations and the online HOMA calculator (available at https://www.dtu.ox.ac.uk/homacalculator/). Insulin secretion was assessed in terms of the insulinogenic index, calculated as (insulin at 60 minutes − insulin at 0 minutes)/(glucose at 60 minutes − glucose at 0 minute) (34). The insulin secretion for the given insulin sensitivity was assessed in terms of the insulin disposition index, calculated as the insulinogenic index divided by the reciprocal of the fasting insulin concentration. The areas under the curve (AUCs) for whole capillary blood glucose of the OGTT were calculated using the trapezoid rule. Those under receiver operating characteristic (ROC) curves were calculated using the “lroc” function of Stata. The body mass index (BMI) was calculated as the prepregnancy weight (kilograms) divided by the height (meters) squared. A BMI of <25 kg/m^2^ was considered lean, 25 to 30 was considered overweight, and >30 kg/m^2^ was considered obese.

### Statistical analysis

Associations were tested by linear regression (for continuous-dependent variables) or logistic regression (for binary-dependent variables). All of the analyses involving PAPP-A were adjusted for the number of weeks of pregnancy when the blood samples were taken. Log-transformed data were analyzed in this study when required to normalize the distribution of the residuals, as required for the linear regression. ROC curve equality was tested using Stata’s “roccomp” function. Data are means (95% confidence interval) unless stated otherwise. A *P* value of <0.05 was considered statistically significant throughout. Statistical analyses were performed using Stata 13 (StataCorp, College Station, TX).

## Results

### Association with GDM and gestational hypertension

**Figure 1.**
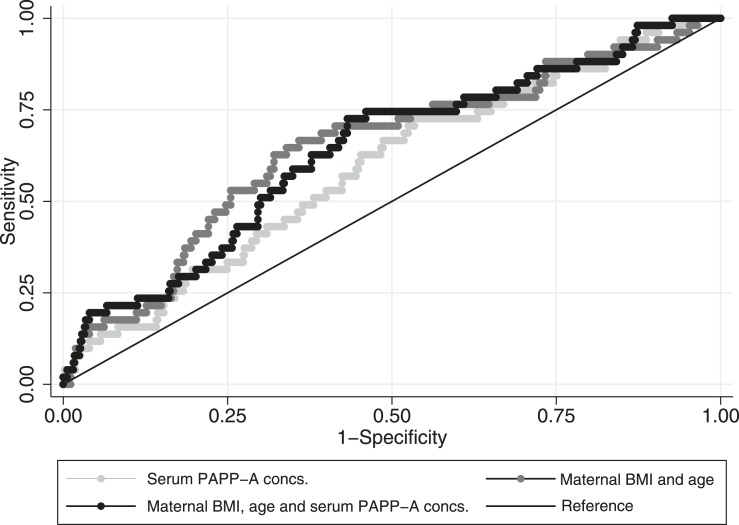
ROC curves for the prediction of GDM development based on: (1) week 15 serum PAPP-A concentrations (AUC = 0.592 ± 0.041), (2) maternal BMI before pregnancy and age (AUC = 0.647 ± 0.042), and (3) a combination of (1) and (2) (AUC = 0.639 ± 0.041). N = 581 per model; AUC data are mean ± standard error of the mean.

Maternal serum PAPP-A concentrations at week 15 were significantly associated with a protective effect on the development of GDM by week 28 of pregnancy [odds ratio (OR) 0.623 (0.453, 0.856), *P* = 3.5 × 10^−3^, McFadden’s pseudo *r*^2^ = 1.7%, n = 777]. The ROC curve AUC was 0.592. Adding PAPP-A to an established model for predicting GDM containing BMI and age did not improve the AUCs (going to 0.639 from 0.647 without PAPP-A; n = 581; *P* = 0.3; Fig. 1). After adjusting for prepregnancy BMI, the statistical significance of the association between GDM and PAPP-A concentrations was lost (*P* = 0.5). Analyzing associations in lean [OR 1.164 (0.677, 1.999), *P* = 0.6, n = 434, of which 24 developed GDM], overweight [OR 0.629 (0.322, 1.228), *P* = 0.18, n = 140, of which 20 developed GDM], and obese [OR 0.545 (0.370, 0.804), *P* = 2.2 × 10^−3^, n = 241, of which 11 developed GDM] women separately, a significant relationship was only seen in obese women. Adjusting the model for fetal sex did not alter the significance of the association between GDM and PAPP-A concentrations [OR 0.617 (0.448, 0.850), *P* = 3.1 × 10^−3^, pseudo *r*^2^ = 1.8%, n = 775], although the relationship was stronger in male [OR 0.575 (0.381, 0.867), *P* = 8.2 × 10^−3^, pseudo *r*^2^ = 2.8%, n = 407] than female [OR 0.670 (0.399, 1.123), *P* = 0.1, pseudo *r*^2^ = 1.8%, n = 368] fetus pregnancies. Associations of maternal serum PAPP-A concentrations at week 15 with the subsequent development of gestational hypertension did not quite reach statistical significance in the number of participants studied [OR 0.613 (0.365, 1.030), *P* = 0.065, McFadden’s pseudo *r*^2^ = 2.1%, n = 361].

### Association with maternal late pregnancy glucose concentrations and indices of insulin secretion and sensitivity in the OGTT

**Figure 2.**
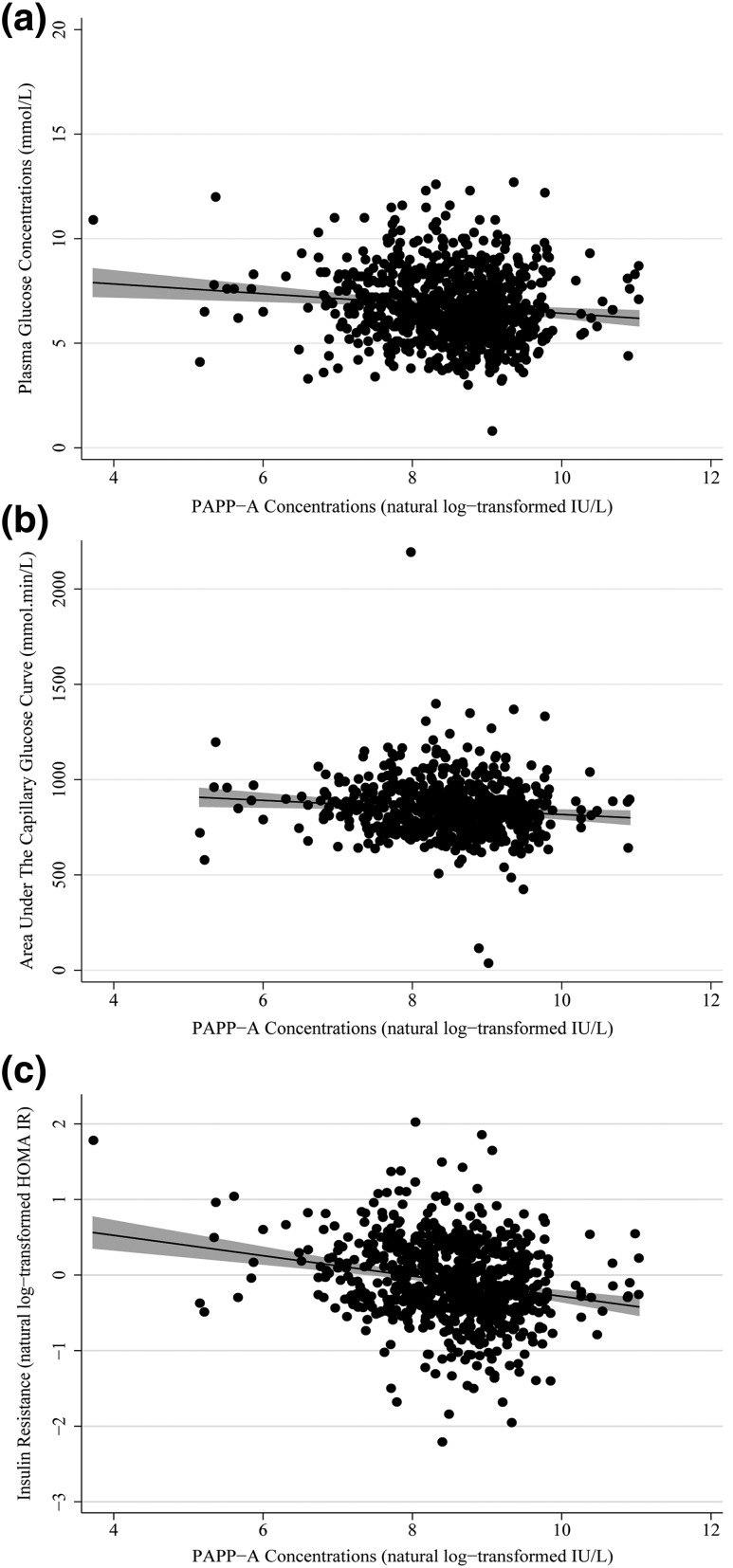
Scatter diagrams illustrate the association between week 15 PAPP-A concentrations and week 28 (a) fasting plasma glucose concentrations, (b) areas under the capillary glucose curve from the OGTT, and (c) insulin resistance (HOMA IR). Also shown are the regression lines and their 95% confidence intervals. All PAPP-A concentrations are natural log-transformed throughout.

Maternal serum PAPP-A concentrations at week 15 were significantly negatively associated with week 28 fasting plasma glucose concentrations (standardized *β* = −0.149, *r*^2^ = 1.2%, *P* = 6.6 × 10^−4^, n = 777) and areas under the capillary blood glucose curve (standardized *β* = −0.144, *r*^2^ = 1.1%, *P* = 3.2 × 10^−3^, n = 610) [Fig. 2(a) and 2(b)]. It was also negatively associated with week 28 HOMA of insulin resistance (IR; standardized *β* = **−**0.319, *r*^2^ = 6.6%, *P* = 1.7 × 10^−13^, n = 768), an association that had the largest effect size [Fig. 2(c)]. This relationship was upheld when the model was adjusted for fetal sex (standardized *β* = −0.324, *r*^2^ = 6.8%, *P* = 8.2 × 10^−14^, n = 766) and when analyzing male fetus (standardized *β* = −0.377, *r*^2^ = 9.0%, *P* = 4.5 × 10^−10^, n = 402) and female fetus (standardized *β* = −0.268, *r*^2^ = 4.5%, *P* = 1.8 × 10^−5^, n = 364) pregnancies separately. Additional negative associations were found between PAPP-A concentrations and plasma glucose concentrations 60 minutes after the 75-g glucose load and the insulin disposition index (Table 2).

**Table 2.** **Association Between Maternal PAPP-A Concentrations at Week 15 [15.0 (14.8, 15.1) Weeks] of Pregnancy and Week 28 OGTT Indices of Maternal Glucose Tolerance and IR and Secretion**

**Index**	**Standardized** **Coefficient (*β*)**	***P* Value**	***r*^2^ (%)**	**n**
Plasma glucose concentration 60 minutes after a 75-g glucose load	−0.188	1.5 × 10^−5^	2.4	777
HOMA insulin sensitivity (HOMA S)	0.319	1.8 × 10^−13^	6.6	768
Insulinogenic index	−0.024	0.6	0	731
Insulin disposition index	0.202	6.5 × 10^−6^	2.6	731

### Association with mean arterial blood pressure in pregnancy

Maternal serum PAPP-A concentrations at week 15 were significantly negatively associated with maternal mean arterial blood pressure at the readings taken around weeks 12, 31, and 37 of pregnancy (Table 3). Maternal mean arterial blood pressures were also significantly negatively associated with week 28 HOMA IR at weeks 12 (standardized *β*-coefficient = −0.219, *P* = 1.9 × 10^−6^, n = 465), 31 (standardized *β*-coefficient = −0.264, *P* = 7.0 × 10^−9^, n = 468), and 37 (standardized *β*-coefficient = −0.318, *P* = 5.7 × 10^−12^, n = 458).

**Table 3.** **Association Between Maternal PAPP-A Concentrations Around Week 15 of Pregnancy and Mean Arterial Blood Pressure Across Pregnancy**

**Pregnancy Stage (Weeks)**	**Standardized** **Coefficient (*β*)**	***P* Value**	***r*^2^ (%)**	**n**
11.8 (11.5, 12.0)	−0.187	4.1 × 10^−3^	1.6	352
31.4 (31.3, 31.5)	−0.202	1.7 × 10^−3^	2.3	355
37.0 (36.9, 37.0)	−0.177	6.9 × 10^−3^	2.5	347

### Association of maternal PAPP-A status with circulating IGF bioactivity

Those women with the highest serum PAPP-A concentrations at week 15 had lower prepregnancy BMIs, were more insulin sensitive at week 28 than those with the lowest PAPP-A concentrations, had slightly lower fasting glucose concentrations at week 28, and had lower mean arterial blood pressures in the second half of pregnancy (Table 4). There was no detectable difference in circulating bioactive IGF activity however.

**Table 4.** **Comparison of the Groups Selected as Having the Highest and Lowest Unadjusted Week 15 Serum PAPP-A Concentrations in Terms of Clinical Characteristics, Week 28 OGTT Indices of Maternal Glucose Tolerance, Insulin Secretion, and Insulin Sensitivity Plus Blood Pressures**

**Index**	**Pregnancy Stage (Weeks)**	**Lowest PAPP-A Concentrations**	**Highest PAPP-A Concentrations**	**Standardized Coefficient (*β*)**	***p* Value**
Maternal age, decimal y	Birth of baby	32.7 (31.4, 34.1)	33.0 (31.6, 34.3)	0.027	0.8
		(n = 48)	(n = 48)		
Parity	Throughout	1.8 (1.5, 2.1)	1.6 (1.3, 1.8)	−0.130	0.2
		(n = 48)	(n = 48)		
Prepregnancy BMI, kg/m^2^	0	27.1 (25.4, 28.7)	22.0 (20.2, 23.8)	−0.442	1.0 × 10^−4^
		(n = 39)	(n = 33)		
Percentage giving birth to males	Birth of baby	54.5	51.1	N/A	0.7
Percentage smoking	At any time during pregnancy	8.9	4.5	N/A	0.4
Percentage who develop GDM	>20	16.7	4.2	N/A	0.09
PAPP-A, mU/L	15	1096 (906, 1326)	18,126 (14,983, 21,929)	0.836	2.0 × 10^−31^
		(n = 48)	(n = 48)		
Bioactive IGF (IGF-IR activation, µg/L	15	2.57 (2.32, 2.81)	2.68 (2.44, 2.92)	0.070	0.5
		(n = 44)	(n = 46)		
Fasting plasma glucose, mg/dL	28	79 (77, 83)	76 (74, 79)	−0.234	0.02
		(n = 48)	(n = 48)		
Plasma glucose 60 minutes after a 75-g glucose load, mg/dL	28	132 (123, 141)	124 (115, 133)	−0.103	0.3
		(n = 48)	(n = 48)		
AUC of capillary whole blood glucose concentrations, g⋅min/dL	28	15.77 (15.03, 16.49)	15.32 (14.53, 16.09)	−0.100	0.4
		(n = 48)	(n = 48)		
Insulinogenic index, Δins_60_/Δgluc_60_	28	148 (122, 179)	144 (118, 174)	−0.024	0.8
		(n = 48)	(n = 48)		
Insulin disposition index, L/mmol	28	13,135 (10,518, 16,403)	17,456 (13,978, 21,798)	0.188	0.08
		(n = 48)	(n = 48)		
HOMA IR	28	1.14 (1.00, 1.30)	0.83 (0.73, 0.99)	−0.331	1.0 × 10^−3^
		(n = 48)	(n = 47)		
Mean arterial blood pressure, mm Hg	12	84 (78, 90)	79 (75, 84)	−0.214	0.4
		(n = 16)	(n = 26)		
	31	89 (85, 94)	78 (75, 82)	−0.522	5.9 × 10^−4^
		(n = 16)	(n = 24)		
	37	91 (86, 96)	83 (79, 87)	−0.398	0.01
		(n = 16)	(n = 24)		

Data are mean (95% confidence intervals).

Abbreviations: Δins_60_, change in plasma insulin concentrations during the first hour of the OGTT; Δgluc_60_, change in plasma glucose concentrations during the first hour of the OGTT; N/A, not applicable.

### Association with baby’s birth weight

Maternal serum PAPP-A concentrations at week 15 were not significantly associated with either the baby’s unadjusted birth weight (standardized *β* = −0.005, *P* = 0.9, n = 772) or with birth weight adjusted for gestational age at birth, sex, and parity (standardized *β* = 0.009, *P* = 0.8, n = 768). However, there was a significant positive association when adjustment was made for gestational age at birth, sex, maternal weight prior to pregnancy, parity, smoking, and gestational age when the blood samples were taken (standardized *β* = 0.112, *P* = 8.4 × 10^−3^, n = 642).

## Discussion

In this study we have confirmed negative associations between early pregnancy serum PAPP-A concentrations and subsequent development of GDM and high blood glucose concentrations. However, our ROC curve analyses suggest that such PAPP-A measurements do not offer an advantage over established risk factors for the prediction of GDM development. Subgroup analysis revealed that the negative association between GDM and PAPP-A was only detectable in obese women and was stronger in male fetus pregnancies. It is difficult to know if this reflects a physiological relationship in obese women/male fetus pregnancies or just a lack of statistical power in the other groups. The negative association between PAPP-A concentrations and gestational hypertension, as observed in other studies (13, 19), almost reached statistical significance in our study (presumably the reason that it did not was due to insufficient statistical power in the lower number of pregnancies studied). Negative associations with mean arterial blood pressures were, however, observed. Uniquely, this study showed that the strongest association between early pregnancy PAPP-A concentrations and an index from the week 28 OGTT was with HOMA IR, suggesting that the principal reason PAPP-A is related to the future development of GDM and high blood pressures is via regulation of insulin sensitivities (with high circulating glucose concentrations and blood pressures both being related to reduced insulin sensitivity). As GDM is related to relative reductions in both insulin secretion and insulin sensitivity, it is also not surprising that another relationship was observed between the early pregnancy PAPP-A concentrations and the insulin disposition index, although the strongest relationship by far remained the one with insulin sensitivity.

The role of circulating PAPP-A in pregnancy appears to be cleavage of certain IGFBPs. Given that circulating free (35) as well as total (36) IGF-I appears to influence insulin sensitivity, we hypothesized that the strong associations observed with GDM, high blood pressures, and insulin sensitivity are underpinned by PAPP-A regulating IGF bioavailability in pregnancy, which in turn regulates insulin sensitivity and ultimately contributes to protection against, or the development of, certain adverse conditions of pregnancy. However, we could not detect a difference in IGF bioactivity between women with some of the highest week 15 circulating PAPP-A concentrations and women with some of the lowest, despite having large differences in week 15 PAPP-A concentrations and week 28 insulin sensitivities. One possible explanation of this is the lack of statistical power available to us in trying to detect a difference in IGF bioactivity in only 96 samples. Alternatively, PAPP-A may affect localized rather than circulating IGF-I and IGF-II concentrations (2). The physiology of the IGF axes and insulin sensitivity is complicated due to the presence of IGFBPs and differences between localized and circulating IGF concentrations. One previous pregnancy study failed to find a relationship around week 28 between either serum total IGF-I or IGF-II concentrations and insulin sensitivity (37), whereas another more recent study found a negative association between week 28 serum total IGF-I concentrations and insulin sensitivity (38). A further study found higher weeks 24 to 28 total IGF-I concentrations in women with GDM (39), possibly resulting from these women having higher placental growth hormone drive, which could lead to reduced insulin sensitivity despite higher circulating IGF-I concentrations (40).

A number of other studies (16, 22–28) have found positive associations between early PAPP-A and the baby’s birth weight, although this finding is not universal (18, 29). Indeed, we failed to find an association when analyzing raw or minimally adjusted data. However, close inspection of the other studies reveals that finding such an association depends on whether PAPP-A levels were analyzed in terms of multiples of the median or just unadjusted concentrations. When we adjusted our PAPP-A concentrations for as many of the factors as that are used in the calculation of multiples of the median as we had available to us, the association with baby birth weights emerged. This was not surprising given that in our cohort the week 28 mother’s insulin sensitivity and glucose concentrations, both of which were associated with week 15 PAPP-A concentrations, were related to the baby’s birth weight.

Although reasonable in size, this study has a number of limitations. First, the number of blood pressure readings available to us was relatively modest, and we could not confirm the association between early pregnancy circulating PAPP-A concentrations and the development of gestational hypertension. That circulating PAPP-A concentrations had significant associations with blood pressures throughout pregnancy suggests that the study was underpowered to find such an association with gestational hypertension (with dichotomous variable analyses having less statistical power than analyses using continuous variables). However, the association with blood pressures tempers this limitation somewhat, and there was sufficient power to detect an association between gestational hypertension and insulin sensitivity. Second, the PAPP-A and bioactive IGF concentration measurements were not done in the same sample as those used for the insulin and glucose concentrations. We do not know whether the week 28 PAPP-A and bioactive IGF concentrations track those at week 15. However, the study was designed to enhance previous findings showing that first trimester PAPP-A concentrations are negatively related to the subsequent development of GDM, which has been achieved. Given the strength of the various associations it is possible that the week 15 and 28 results would be related, albeit probably imperfectly (8). The final limitation is that the study was designed to replicate first trimester findings and, technically, the stage of pregnancy when the blood samples were adjusted to, at week 15, was in the second trimester. Although PAPP-A values would probably have been higher at week 15 than earlier in pregnancy (8), the factors that affect circulating PAPP-A concentrations in pregnancy, apart from gestational age and maternal weight and height, tend to be fixed rather than fluid (8), so the association trends are unlikely to have been affected.

In summary, we confirmed associations between circulating PAPP-A concentrations in week 15 of pregnancy and the future development of GDM and high blood pressure. Underpinning these associations was an even stronger association with insulin sensitivity, which may relate to the cleavage of IGFBPs by PAPP-A, leading to increases in bioactive IGF which regulates insulin sensitivity, at least outside pregnancy (35, 36). Further studies on the effect of IGFs on insulin sensitivity in pregnancy would therefore appear warranted.
